# 

**Published:** 1896-12

**Authors:** 


### CONTENTS:

### SELECTIONS.

Article I.—Dental and Medical Infection. - - - - - 337
" II.—The Army Medical Museum and Library. Dr. D. L. Huntingdon. - - - - - - - 340
" III.—Novel Operation on the Eye. - - - - - 349
" IV.—The Life and Growth of the Tooth. Dr. D. D. Smith. 352
" V.—A Plan for a National Dental Association Divided into Four Co-ordinate District Branches. A. H. Thompson, D. D. S. - - - - - - 354
" VI.—Suggestions of Crown Work. H. W. Allwine, D. D. S. 357
" VII.—The Dental Society of the State of New York. - 359
" VIII.—The Physical Characteristics of the Teeth. Thos. Fletcher, D. D. S. - - - - - - 375

### MONTHLY SUMMARY.

Treatment of Antrum Disease. - - - - - - 377

A Valuable Hint. - - - - - - - - 378

A Word of Caution. - - - - - - - 378

Interesting Experience. - - - - - - - 379

New Local Anæsthetic. - - - - - - - 379

Congenital Teeth. - - - - - - - - 380

Influence of Condiments on Digestion. - - - - 380

Why Amalgams Fail. - - - - - - - 381

Toothache Drops. - - - - - - - - 381

White Caries. - - - - - - - - 382

Peculiar Affection of the Mucous Membrane of the Lips and Mouth. - - - - - - - 383

Investing Material. - - - - - - - 383

Death Three Days after Chloroform Administration. - - 384

Treatment of Pulpless Teeth. - - - - - - 384

Dental Inspectors for Schools. - - - - - - 384

